# Inter-laboratory reproducibility of an untargeted metabolomics GC–MS assay for analysis of human plasma

**DOI:** 10.1038/s41598-020-67939-x

**Published:** 2020-07-02

**Authors:** Yanping Lin, Gary W. Caldwell, Ying Li, Wensheng Lang, John Masucci

**Affiliations:** Janssen Research and Development, Johnson and Johnson, 1400 McKean Road, Spring House, 19477 PA USA

**Keywords:** Biomarkers, Metabolomics

## Abstract

There is a long-standing concern for the lack of reproducibility of the untargeted metabolomic approaches used in pharmaceutical research. Two types of human plasma samples were split into two batches and analyzed in two individual labs for untargeted GC–MS metabolomic profiling. The two labs used the same silylation sample preparation protocols but different instrumentation, data processing software, and database. There were 55 metabolites annotated reproducibly, independent of the labs. The median coefficient variations (CV%) of absolute spectra ion intensities in both labs were less than 30%. However, the comparison of normalized ion intensity among biological groups, were inconsistent across labs. Predicted power based on annotated metabolites was evaluated post various normalization, data transformation and scaling. For the first time our study reveals the numerical details about the variations in metabolomic annotation and relative quantification using plain inter-laboratory GC–MS untargeted metabolomic approaches. Especially we compare several commonly used post-acquisition strategies and found normalization could not strengthen the annotation accuracy or relative quantification precision of untargeted approach, instead it will impact future experimental design. Standardization of untargeted metabolomics protocols, including sample preparation, instrumentation, data processing, etc., is critical for comparison of untargeted data across labs.

## Introduction

Metabolomics has become essential for understanding the impact of external or pathological stressors on a biological system^1^. In the last decade, both targeted and untargeted metabolomics have shown great potential in phenotyping metabolites changes and providing collective information on the end results of normal physiology and on diverse pathophysiological stimuli in tissues, cells, and biofluids. From a drug development perspective, the goal of system biology is to integrate the “-omics” approaches with biological-computational methodologies to increase the success rate in selection of drug candidate and clinical indication^2^. While targeted metabolomics has an established role as a valuable decision-making tool in drug discovery and development, it is essential to reveal what role of untargeted metabolomics plays, especially regarding its long-concerned reproducibility issues. With the development of new technologies, recent studies have transitioned metabolomics from proof-of-principle to validation^3,4^. In these studies, untargeted metabolomics allowed a hypothesis to be generated and challenged to validate new biomarkers of diseases^5–7^.

However, the implementation of untargeted metabolomics needs to be validated due to the concerns on repeatability of the method. A typical workflow of untargeted metabolomics includes sampling, extraction procedures, instrument setting, data processing, statistical screening and biological interpretation. Theoretically, each step along the workflow can introduce artifacts into the results. There is nice overview papers summarizes the challenges of conducting untargeted metabolomic research and they also recommended possible strategies to minimize the variations^8^. Several inter-laboratory studies have attempted to validate the accuracy of the metabolomic approaches, either using Nuclear Magnetic Resonance (NMR) of different magnetic fields^4,9^, Gas Chromatography–Mass Spectrometry (GC–MS)^10,11^ or Liquid Chromatography-Mass Spectrometry (LC–MS)^12^. However, they did not address comparisons of heterogeneous instruments or methods nor the fact that strict protocol designs are difficult to extrapolate to real-life situations.

Thus, to respond to the inter-laboratory reproducibility challenges and to facilitate standardization initiatives, it would be wise to determine current variations in untargeted metabolomics assays, especially cross laboratory results using heterogeneous instruments. Especially for researchers who need to out-source their studies to available efforts, it would be nice to give a plain example to shape their expectations and prepare their next steps. Compared to LC–MS untargeted metabolomic assays, GC–MS assays have solid retention time and relative mature database for identification. In this study, we conducted interlaboratory comparison with the untargeted metabolomic assays using GC–MS, specifically accessing the repeatability from the two perspectives of its outcome annotation and ion intensities of specific ions. Our primary goal was not to claim which lab’s assay was more accurate, instead we intended to point out the possible sources of variations of interlaboratory assays and initiated the awareness and efforts to reduce it.

## Results

### Annotation repeatability

#### NIST samples

**Figure 1:**
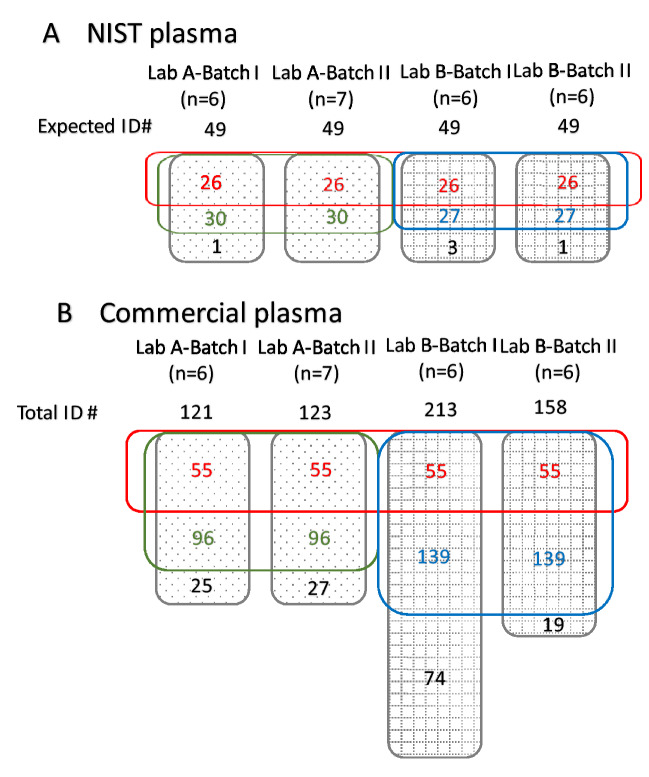
Illustration of annotation numbers in Lab A and Lab B from (**A**) NIST plasma and (**B**) commercial plasma. Specifically: (**A**) There are 49 known metabolites spiked in NIST plasma detectable by GC–MS. Lab A commonly annotated 30 metabolites in two batches (green rectangle); Lab B commonly annotated 27 metabolites in two batches (blue rectangle); Lab A and Lab B commonly annotated 26 metabolites in all batches (red rectangle). (**B**) Lab A commonly annotated 96 metabolites in two batches (green rectangle); Lab B commonly annotated 139 metabolites in two batches (blue rectangle); Lab A and Lab B commonly annotated 55 metabolites in all batches (red rectangle).

There were two types of human plasma samples tested by Lab A and Lab B, each type analyzed in two batches Batch I and Batch II separately. One type of human plasma samples was SRM1950 purchased from National Institute of Standard and Technology (NIST), which was fortified known metabolites in certain concentrations. Based on the fact sheet released by NIST^13^, there were 49 metabolites with fortified concentrations detectable by GC–MS. The annotation repeatability of two labs for NIST sample is demonstrated in Fig. 1A. After the untargeted metabolomic profile approaches, after two batches, Lab A and Lab B annotated 30 and 27 metabolites respectively. Although Lab B had slightly less annotations compared to Lab A, they still had 26 overlapped metabolites where majority of them were amino acids. Within Lab A, 30 metabolites were repetitively annotated, except behenic acid which was only included in Batch I. Within Lab B, compounds arachidic acid, arachidonic acid, and leucine were identified only in Batch I and cysteine was only annotated in Batch II. The repetitive annotations are listed in Table 1.

**Table 1:** Common Annotations in Commercial and NIST Plasma Samples Shared by Lab A and Lab B.

#	Metabolites Name	Pubchem ID	NIST Included^a^	#	Metabolites Name	Pubchem ID	NIST Included^a^
1	1-Monostearin	24,699		29	Maltose	6,255	
2	Aconitic acid	444,212		30	Mannitol	6,251	
3	Alanine	5,950	Yes	31	Methionine	6,137	Yes
4	Alpha-ketoglutarate	51		32	Myo-inositol	892	
5	Aspartic acid	5,960		33	Myristic acid	11,005	Yes
6	Beta-alanine	239		34	Oleic acid	445,639	Yes
7	Capric acid	2,969		35	Oxalic acid	971	
8	Cholesterol	5,997	Yes	36	Oxoproline	7,405	
9	Citric acid	311		37	Palmitic acid	985	Yes
10	Citrulline	9,750		38	Palmitoleic acid	445,638	Yes
11	Creatinine	588	Yes	39	Pelargonic acid	8,158	
12	Ethanolamine	700		40	Phenylalanine	6,140	Yes
13	Glucose	5,793	Yes	41	Phosphate	1,004	
14	Glutamic acid	33,032		42	Phthalic acid	1,017	
15	Glutamine	5,961		43	Proline	145,742	Yes
16	Glycerol	753		44	Pyruvic acid	1,060	
17	Glycine	750	Yes	45	Quinic acid	6,508	
18	Glycolic acid	757		46	Ribose	5,779	
19	Heptadecanoic acid	10,465	Yes	47	Serine	5,951	Yes
20	Hypoxanthine	790		48	Stearic acid	5,281	Yes
21	Indole-3-acetate	802		49	Threonine	6,288	Yes
22	Isoleucine	6,306	Yes	50	Tryptophan	6,305	
23	Lactic acid	612		51	Tyrosine	6,057	Yes
24	Lauric acid	3,893	Yes	52	Urea	1,176	Yes
25	Levoglucosan	2,724,705		53	Uric acid	1,175	Yes
26	Linoleic acid	5,280,450	Yes	54	Valine	6,287	Yes
27	Lysine	5,962	Yes	55	Xylose	135,191	
28	Lyxitol	439,255					

^a^There are two more metabolites, leucine and arachidic acid commonly annotated in only NIST samples by Lab A and Lab B.

#### Pooled commercial human plasma

Another type of human plasma tested for annotation repeatability was pooled gender human plasma, purchased from BioIVT (Westbury, NY). There were 55 metabolites commonly annotated by Lab A & Lab B cross two batches. The annotation results of commercial human plasma were presented in Fig. 1B. Within Lab A, there are 96 components, 78% of the annotations repetitively showing between the 121 of Batch I and 123 of Batch II, correspondingly. Although there were 139 common annotations within Lab B between two batches, the variation of annotating amounts was much larger in Lab B compared to that of Lab A considering the 213 and 158 annotations for Batch I and Batch II, correspondingly.

The variations of annotated numbers are reasonable, considering there could be many factors lead to such variations in annotations. These reasons might include but not limited to, how the spectra were deconvoluted from collected signal, how they were grouped and aligned as spectra for one potential compound, and how the database searching algorithm was set up. Even all these factors were kept consistent in the same lab, the raw file collected by instrument fluctuate dynamically.

#### Network mapping based on common annotation

**Figure 2:**
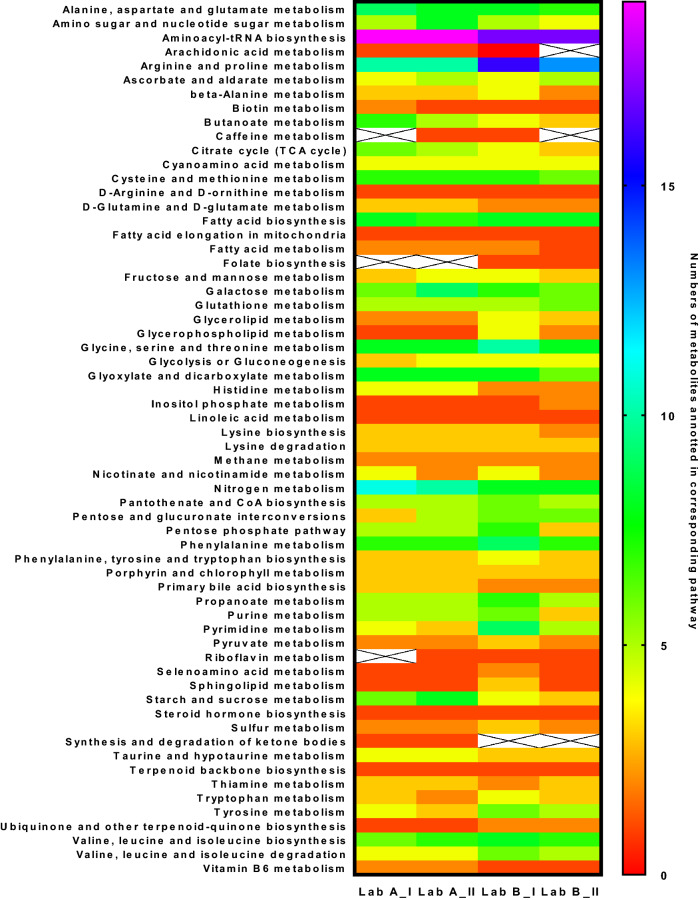
Heat map of the repeatability of revealed metabolic pathways across Lab A and Lab B. X-axis: sample batches, Left Y-axis: metabolic pathway, color: numbers of metabolites annotated in certain pathway.

The annotated metabolites in each batch were submitted to network mapping to investigate their abilities in revealing metabolic pathways. The results are presented in the heatmap in Fig. 2. Generally, the annotated metabolites pointed to 62 metabolic pathways. Majority of the metabolic pathways were described by around 5 metabolites. Although Lab A and Lab B had 96 and 139 common annotations correspondingly, there was no significant difference of their abilities in directing to metabolic pathways. The pathway named “Synthesis and degradation of ketone bodies” was unique in Lab A, which was linked by only one metabolite annotation. The unique pathway named “folate biosynthesis” revealed only by Lab B was also related with only one metabolic annotation. That means among all the metabolites involved in folate biosynthesis pathway, only one metabolite was potentially annotated by Lab B results. Whether this annotation is true metabolite identification needs further works to prove.

**Figure 3:**
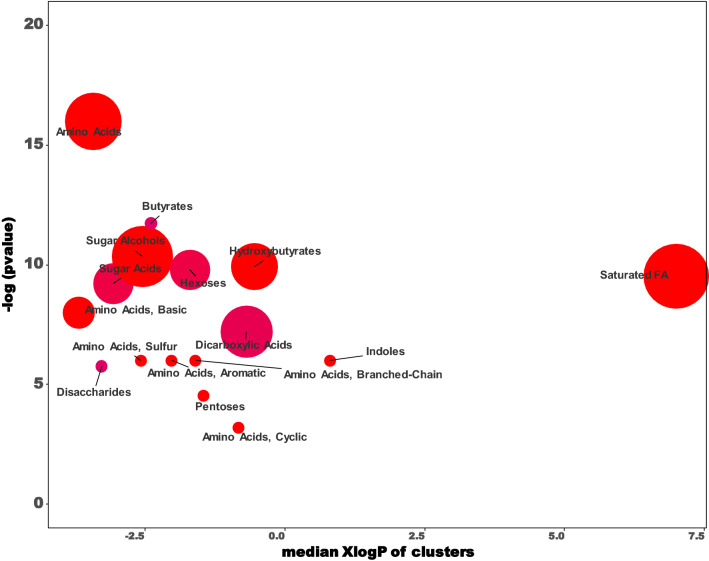
Cluster plot of the overlapped annotations from Lab A Batch I & II. The annotated metabolites were clustered by their structural similarity. A student t-test was conducted between the ion intensities of NIST and pooled human plasma samples. X-axis: polarity of metabolites, Y-axis: significance of the difference, log transformed p-value of the student t-test. Red: pooled human plasma compared to NIST increased, blue: pooled human plasma compared to NIST decreased, dot size: the more metabolites clustered the bigger.

It is obvious that not every metabolite in plasma could be identified by untargeted metabolic approaches due to detection limitations. Based on the structural similarity of the annotated metabolites (commonly annotated in Lab A), they were clustered to reveal the detecting capability of the untargeted GC–MS approach. The cluster plot of the repetitive annotations in Lab A is presented in Fig. 3, which showed that sugar alcohols, sugar acids, amino acids, dicarboxylic acids and some saturated fatty acids were the dominant metabolites annotated by the untargeted GC–MS approach. Koek et al. had reviewed in detail that what types of metabolites are able to be annotated by untargeted GC–MS metabolomic approach^8^. Our results could by another example for their statements.

### Ion intensities

#### Absolute ion intensities of FAMEs ladder

The fatty acid methyl esters (FAMEs) mixture contains C8, C9, C10, C12, C14, C16, C18, C20, C22, C24, C26, C28, and C30 linear chain length, resulting in a serious of retention times like a “ladder “distributed throughout the run-time. Thus, FAMEs work as a mixture of internal retention index (RI) markers to test metabolites derivatized by *N*-Methyl-*N*-(trimethylsilyl)trifluoroacetamide (MSTFA). The same amount of FAMEs was spiked in each sample in both labs, thus they also play the role as “cross-lab internal standards” in our study just like isotopic materials in classic quantitative studies. Because of the included retention index information, the corresponding spectra libraries, are better suited for unambiguous compound identification than smaller libraries or mass spectral libraries that lack consistent retention information. This system was first developed by Dr. Oliver Fiehn^14^ and commercialized by Agilent^15^ applied to the untargeted metabolomic profiling for plasma samples using GC–MS.

**Figure 4:**
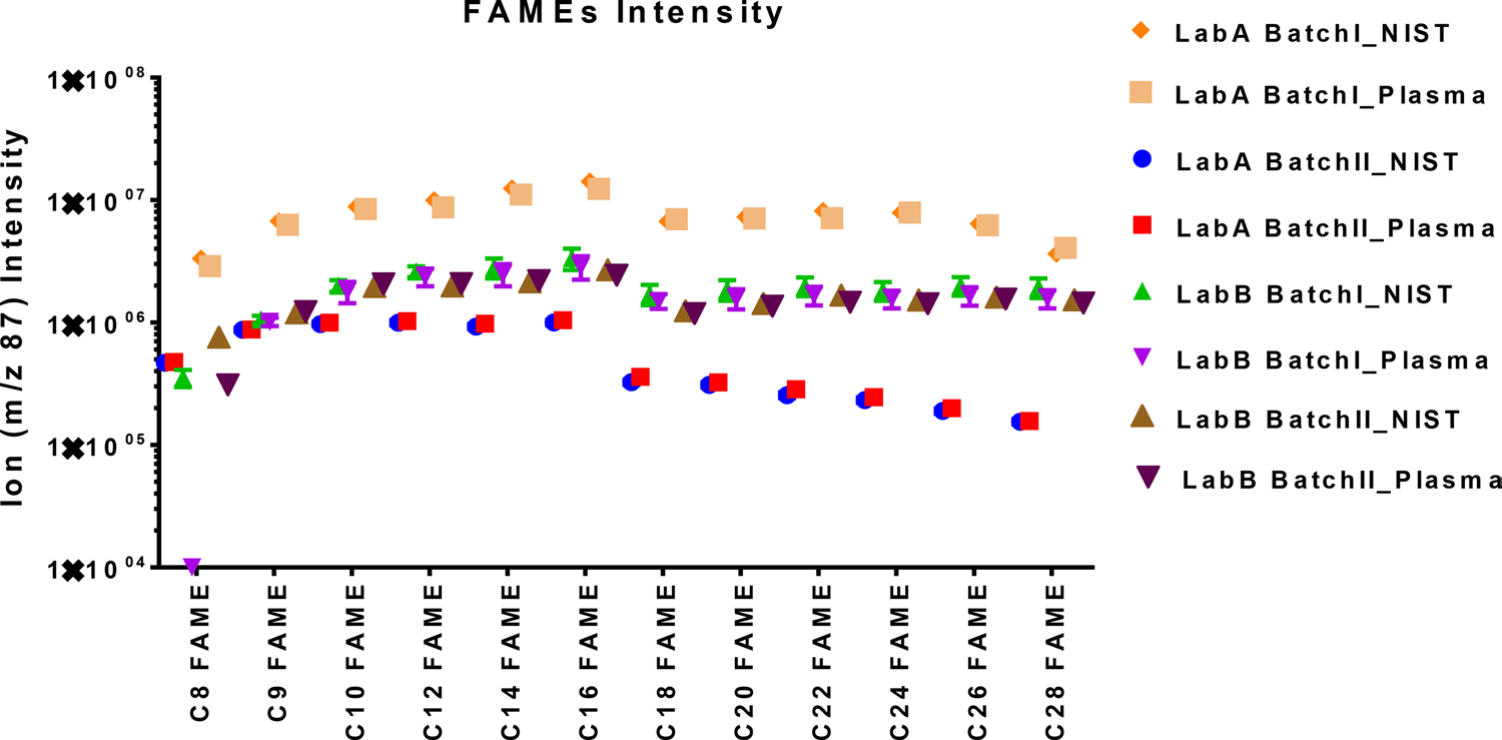
Absolute ion intensities of the same concentrations of FAMEs ladders spiked in every sample analyzed in Lab A and Lab B. Due to instrument fluctuation, there were variations of the absolute ion intensity of the same levels of FAMEs standards cross batches inter-or intra-laboratory.

Since both Lab A and Lab B used the same sample preparation protocol, the FAMEs and MSTFA silylation system with corresponding database, the same amount of FAMEs ladders serving as retention time lock reagent existed in each batch of data. FAMEs are great markers to indicate the ion intensity variations in multiple runs. The absolute ion intensities of the quantification mass to charge of FAMEs (m/z 87) are shown in Fig. 4. Due to the instrumentation fluctuation, the absolute ion intensities of the same concentrations of FAMEs in each sample fluctuated among batches inter- or intra- laboratory. Meantime, the ion intensities within the same analytical batch either in commercial plasma or NIST plasma were overlapped with each other. This phenomenon proved that each batch, both the sample preparation and instrument status were at a qualified level and stable across samples. Thus, the ion intensities of FAMEs ladder in each batch could be considered as “internal standard” to normalize each individual sample within this batch.

Meanwhile, the medians of coefficient of variation (CV%) of absolute ion-intensity were listed in Table 2. Since in this study the NIST plasma and pooled commercial plasma were assumed as biological different groups, the analysis of their precision, revealed as CV% were calculated separately. Furthermore, the FAMEs ladders were consistent “internal standards” in each sample, so they were separated out from all the other annotations. As shown in by the median CV%, the ion intensities of Lab A were more precise compared to that in Lab B. Within lab, there could be difference of the CV% cross batches, such as Lab A Batch I and Batch II. Despite the difference, Lab A generally had very decent precision, < 15%, which might be attributed to the high-resolution mass spectrometry used in Lab A. The precisions of FAMEs ladder were always better compared to those of the annotated metabolites, which was independent of the labs. It makes sense, since the FAMEs ladder was known components precisely spiked into each sample. The CV% of the annotated metabolites represented the actual spectra performances during the untargeted GC–MS run.

**Table 2:** Median of the coefficient variants (CV%) describing absolute spectra ion intensity in Lab A and Lab B.

	Lab A		Lab B	
	NIST (%)	Plasma (%)	NIST (%)	Plasma (%)
**FAMEs ladders (n = 14)**				
Batch I	15.0	9.1	16.8	16.2
Batch II	6.1	4.1	17.5	17.1
**All annotated metabolites**				
Batch I	15.3	14.1	19.1	26.1
Batch II	12.7	13.1	21.5	30.3

#### Relative ion abundance

As clearly showed by FAMEs data, the absolute ion intensity of the same component was not always consistent among batches and cross labs. This change in ion intensity is major reasons to normalize data before comparing different group of samples to select distinguished metabolites as potential biomarkers. In this study we used the averaged FAMEs ion intensity in each sample as the denominator to normalize each metabolite annotated in the corresponding sample. In other words, we used an average normalization protocol to adjust the absolute ion intensity at the sample level to get relative ion intensities.

**Figure 5:**
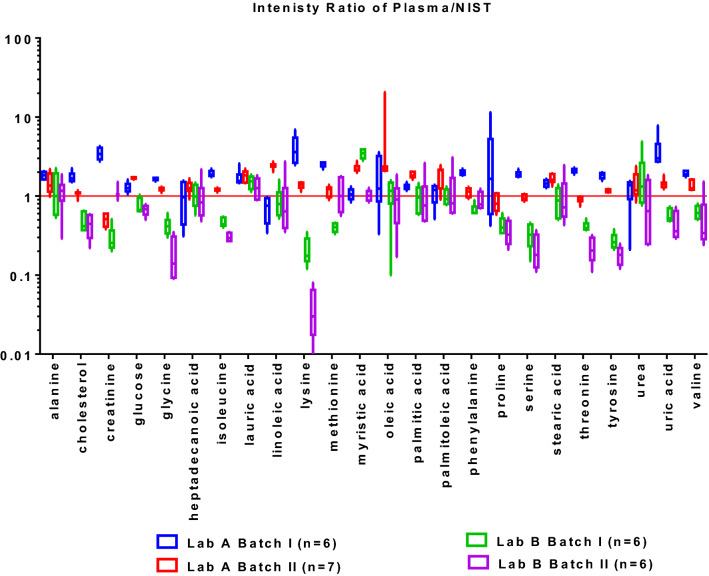
After normalization to the average FAMEs’ ion intensity, the ion intensities of commonly identified metabolites in commercial plasma were divided by those in NIST correspondingly. Box represents the 95% confidence interval of six replicates of the relative ion intensity ratios of plasma/NIST; Middle line: the average of the ion intensity ratio of plasma/NIST; top and bottom lines; the maximum and minimum values of the ion intensity ratios of plasma/NIST.

The following question would be whether relative ion abundance is reproducible cross labs. Because the typical working flow is comparing the relative ion abundance among biological groups to screen out metabolic changes. We found out the commonly annotated metabolites in commercial and NIST plasma within the same batch. After normalization, the relative ion intensities of these common metabolites in commercial plasma were divided by those in NIST plasma correspondingly. Figure 5 presents the comparison of these ion ratios. A ratio above 1 means the concentration of the metabolite in commercial plasma is higher compared to that in the NIST sample; the opposite means the concentration of this specific metabolite was higher in the NIST. Many of these metabolites were determined by Lab A having higher concentrations in commercial plasma. While according to lab B, many of them are higher in NIST plasma. Specifically, nine out of the plotted 24 metabolites (37.5%) specifically including cholesterol, glucose, glycine, isoleucine, lysine, phenylalanine, serine, uric acid and valine had opposite ratios between Lab A and Lab B. Occasionally, the ion intensity ratios of some metabolites like creatinine and linoleic acid were not consistent between batches within the same lab. The variations of normalized relative ion intensities in Lab B were slightly larger compared to that of Lab A. This might be related to the lower resolution of mass spectrometry used by Lab B (unit resolution) compared to Lab A (high resolution).

### Power analysis

Statistical power analysis relates sample size, effect size, and significance level to the chance of detecting an effect in a data set. In power analysis, the effect size corresponds to the quantitative measure of the strength of a phenomenon relative to the variation in the population (e.g., how different two groups of samples are relative to the within- group variance). The stronger the effect, the more easily it will be detected, thus requiring a smaller number of samples to meet similar power requirements^16^. Power analysis is normally performed before the beginning of a study, working as a safeguard that estimates the probability of obtaining meaningful results and thus success of a study.

Here, we performed the power analysis using the absolute spectra ion intensity of the annotated metabolites after data-acquisition as an evaluator to indicate the impact of inter-laboratory variations of the workflow. Furthermore, the spectra intensity dataset went through various sample normalization, data transformation and data scaling tests to evaluate the potential impacts of post-acquisition data processing manners on predicted power. The predicted power (maximum as 100%) of Lab A Batch II (123 features of 14 samples) and the Lab B Batch I (213 features of 12 samples) were listed in Table 3. Surprisingly, without any post-acquisition data processing, Lab B had higher predicted power compared to lab A, which might be due to more features (213 compared to 123) in the raw data set. For both labs, the best normalization method taking the raw spectra intensity normalized by that of FAME C14. Data transformation method was data set dependent. For lab A, log transformation was more proper, while for lab B, we needed the cube root of each data to better fit normal distribution. It seemed data scaling did not impact predicted power at all, which would be a totally different case in multivariate analysis, such as principle component analysis (PCA), partial least square (PLS) and so on^17,18^. We also calculated how many samples per group would be needed to reach a predictive power above 0.80 (80%). The results indicated that for a dataset like Lab A Batch II, we need 200 sample per group, while dataset like Lab B Batch I needs 40 samples per group. That might be attributed to larger CV% of Lab B compared to Lab A. Since NIST and commercial plasma are assumed different biological groups, they are similar. It makes sense that the large CV% in Lab B dataset was mistakenly taken as the “large biological variance” in the power analysis leading to a smaller sample size. This results seems to us that it won’t harm to conduct power analysis after study to get a better sense of the quality of data and help plan next steps, although power analysis is normally recommended to be performed before the beginning of the study to get a better experimental design.

**Table 3:** Predicted power (maximum as 1) of Lab A Batch II (n = 7 per group, NIST and plasma groups) and Lab B Batch I (n = 6 per group, NIST and plasma groups) dataset with and without normalization, data transformation and data scaling.

	LabA_BatchII		LabB_BatchI	
	n = 7 per group	If n = 200 per group	n = 6 per group	If n = 40 per group
Raw data set	0.23	0.85	0.46	0.85
Normalization by sum	0.27	0.83	0.23	0.67
Normalization by median	0.14	0.8	0.43	0.85
Normalization by reference feature^a^	0.27	0.87	0.48	0.87
Quantile normalization	0.23	0.82	0.44	0.85
Log transformation	0.37	0.87	0.38	0.74
Cube root transformation	0.26	0.84	0.42	0.84
Mean centering	0.23	0.85	0.46	0.85
Auto scaling	0.23	0.85	0.46	0.85
Pareto scaling	0.23	0.85	0.46	0.85
Range scaling	0.23	0.85	0.46	0.85
Combine Normalization with data transformation^b^	0.45	0.9	0.49	0.8

^a^The reference feature was the ion intensity of the component of fatty acid methyl esters (FAME) with C14 linear chain length in each sample. Because FAME C14 has a retention time at the middle of run and has decent ion intensities.

^b^For lab A, the best combination is normalization by reference feature and log transformation; for lab B, the best combination was normalization by reference feature and cube root transformation.

## Discussion

Our study was designed to evaluate the ability of untargeted metabolomics approaches to produce convergent results at the metabolic profiling level by heterologous instruments located in different laboratories when analyzing the same samples. The reasons we chose GC–MS over LC–MS for this investigation is due to the easily standardized sample preparation procedure and more mature database development for metabolic annotation. This study has the scope to help us assess to what extent results can be platform independent and what might be the critical points to generate reliable data when there are cross lab collaborations to analyze the same piece of samples.

The general workflow of untargeted metabolomic approach is composed by sampling collection and preparation, instrument operation, data processing, statistical screening and biological interpretation. In this study, Lab A and Lab B followed the same protocol to process the exact same samples. The assumption here was that the variations from sampling and preparation were minimized. Despite the heterologous instruments, subsequently different data processing protocols corresponding to each instrument and searched against different databases, there are still quite number of metabolites (55 metabolites) repetitively annotated cross labs. These platform independent metabolites usually are the commonly accessed metabolites. They are included in many databases, serve in multiple basic metabolic pathways, and many times have higher level in biological matrix. More specifically, these commonly annotated metabolites belong to so called “Class-I metabolites” described in Koek’s review^8^. They are metabolites containing hydroxylic and carboxylic functional groups, such as sugars, organic acids and fatty acids, whose analytical performance is repeatable and intermediate after silylation derivatization. On the other hand, within either lab, cross batches, the reproducibility of annotations was much higher (96 metabolites in Lab A, 139 metabolites in Lab B). Martin et al. also found that a high convergence in the spectral information could be produced instrumental independently if the same set of samples are described^1^. Although there was extra sialylation procedure involved in our study, both Lab A and Lab B followed the same sample preparation procedure and analyzed the same samples. Our study along with others’ findings suggest that the standardized workflow, instrumental operation, data processing and searching against the same database all contribute to reliable and repeatable metabolic annotations.

Another common application for untargeted metabolomic approach is to use the spectral intensity as relative quantification variables to compare metabolic profiles. Potential metabolic “biomarkers” will be screened out after the comparison and then to further correlate with biological status. Our results show that the spectral intensities of the same metabolite are not always consistent across labs, although the metabolites are identified from the same sample prepared by standardized protocol. We’d like to interpret this as the comparison among biological status, for example, disease and healthy groups, based solely on untargeted metabolomic approach for relative quantification is critically relying on the repeatability cross labs. That means without further validation nor strict control of the precision, the correlation established between metabolic fluctuation and biological status, for example metabolite in disease group is much higher than healthy group, is not trustable. From another perspective, this is a good illustration of the necessity of extra care to be implemented in post-data acquisition curation.

One argument might be performing normalization to correct and adjust ion intensities to increase the reliability of relative quantification. There are multiple algorithms for raw spectral intensity normalization, like mixture model normalization using pooled quality control samples^19^, mean centering, median scaling, quartile normalization^20^, EigenMS^21^, batch normalizer^22^, and the sum peak height of FAMEs internal standards (_f_TIC)^11^. There is even an online service enabling performance evaluation of various normalization methods from multiple perspectives, called NOREVA^23^. In this study, we evaluated not only several common normalization strategies, but also other popular post-acquisition data processing manners, including data transformation and data scaling. As presented by Table 3, post-acquisition processing might shift your predicted power. In another word, with the existence of spectra variations, the predictive power might be different than your experimental design even after the correction of post-acquisition of data processing. Meanwhile, unfortunately, normalization could not completely correct the variations generated throughout the untargeted workflow. Figure 5 was generated after normalization in our study. We can still see the inter-laboratory discrepancy of changing trend between two groups (Plasma/NIST) of the commonly annotated metabolomic biomarkers. The normalization we used in this study could make adjustment at sample level. Various normalization techniques used in this field are not only able to adjust each sample, some of the normalization algorithms even can make adjustment for each metabolite. Since the goal of normalization is to reduce the systematic variation but preserve the biological variation, normalization techniques are deemed successful if the variance has decreased^24^. However, some of the important biological variation may have been removed if we over-normalize data. Even the normalized results need to be validated by comparing them to the results from panel of targeted assays. Therefore, it’s not realistic to rely on normalization to correct all the variations in untargeted approach to achieve a repeatable comparison among biological groups.

Further validations are necessary to enhance the repeatability of untargeted metabolomic approach either for metabolic identification or for the assessment of correlations between metabolic fluctuation and biological changes. The most defensive method to confirm a metabolic ID is to use reference standard. Once the mass spectrometry features of the reference standard are consisted with what you have for your metabolic annotation, the identification is confirmed^25,26^. It takes more efforts to confirm the correlations between metabolic fluctuation and biological changes. The validation of relative quantification can be achieved by recruiting more samples, using targeted quantification assay to re-analyze the same piece of samples or comparing to similar studies.

Network mapping is widely applied as the last step of most untargeted metabolomic studies using annotated metabolites or the unique mass spectrometry features. It helps to highlight the most interested pathways for further targeted analysis. However, extra attention should be payed to the biological matrix when conducting network mapping. For example, some metabolic reactions occur at the sub-cellular level^27,28^. Sometimes extra cautions are needed when elucidating pathway flow only based on analytical results from collective biological matrix, such as serum, plasma or urine.

## Conclusion

This study used two GC–MS systems to assess the reproducibility of untargeted metabolomic approaches in obtaining reliable metabolomic profiles. The commonly accessed metabolites could be repetitively annotated, independent of the platform. However, relative quantification is not easily reproducible; thus, in other words, the screened metabolic biomarkers are fluctuating between batches and cross labs. The novelty of the study is that it reveals the numerical details about the variations in metabolomic annotation and relative quantification using plain inter-laboratory GC–MS untargeted metabolomic approaches. Especially we compare several commonly used post-acquisition strategies and found normalization could not strengthen the annotation accuracy or relative quantification precision of untargeted approach, instead it might shift the predictive power of the outcome subsequently twist future experimental design.

Once again, the study suggests that standardization of protocols for untargeted metabolomic working flow is critical for comparison of data across labs. Without standardization, different biological interpretations of the data are highly likely to occur across labs. Further validation for both metabolic annotation and relative quantification are essential to make accurate biological interpretations.

## Materials and method

### Lab A

#### Instrumentation setup

Lab A used a 7890B Agilent GC with LECO Pegasus IV time-of-flight MS instrument (Leco, St. Josph/MI, USA). The column was 30 m long Restek 95% dimethyl/5% diphenyl polysiloxane RTX-5MS column, 0.25 mm internal diameter, 0.25 um film, and a 10 m empty guard column (Restek, Bellefonte, PA). The autosampler was a Gerstel automatic liner exchanger with a multi-purpose autosampler system and a cold injection system (ALEX MPS2/CIS) (GERSTEL, Mulheim an der Ruhr, Germany). Each sample was injected at 0.5 µL through the multi-baffled glass liners (Restek, Bellefont, PA) under splitless mode operation with a 25 s purge time, 40 ml/min purge flow, Helium carrier gas (5.0 grade), and a column carrier gas flow of 1 ml/min. The initial temperature of the injection liner was 50 °C, the equilibration time was 0.5 min, and the temperature increased 12 °C/second to 275 °C with a hold time of 3 min. The oven was initially set at 50 °C held for 30 s, and then ramped at rate of 20 °C/min to a final temperature 330 °C, with a hole time of 10 min.

The transfer line of the time of flight mass spectrometry (TOF–MS) was set at 280 °C, with a solvent delay of 5.6 min. The ion source temperature was 250 °C. Mass Spectra were collected from 85–500 Da at 70 eV electron ionization energy. The scan rate was maintained at 17 spectra per second.

#### Samples

Two types of plasma samples were used in this study for both Lab A & Lab B. One type was purchased from National Institute of Standard and Technology (NIST, https://srm1950.nist.gov/); the other type of human plasma, pooled genders, using EDTA as anticoagulant were purchased from BioIVT (Lot # BRH1542002, Westbury, NY). Materials and methods study protocols and amendments were reviewed by an Independent Ethics Committee or Institutional Review Board, as appropriate, for each lab.

The experimental protocols in Lab A were approved by West Cost Metabolomics Center (Davis, CA, USA) and carried out in accordance with relevant guidelines and regulations. Two batches of NIST (Batch I, n = 6; Batch II, n = 7) and pooled human plasma samples (Batch I, n = 6; Batch II, n = 7), each 30 µl, were kept in a − 80 °C freezer until prepared for GC–MS analysis in November 2017 and May 2018, respectively. Sample extraction and purification protocols followed those outlined in Reference^11^. Briefly, samples were first extracted with a solution mixture composed of isopropanol, acetonitrile and water (3:3:2, v/v/v). These samples were further extracted with acetonitrile and water (50:50, v/v). The supernatant was evaporated to dryness in a SpeedVac system (Thermo Savant SPD 1010). The residue was first oxidized with 10 µL of freshly prepared Methoxyamine hydrochloride (MeOX) solution, 20 mg/ml in pyridine, at 30 °C for 1.5 h on a thermo shaker (Eppendorf, Thermomixer R) at 800 rpm. Then the samples were further derivatized using 90 µl of *N*-methyl-*N*-(trimethylsilyl) trifluoroacetamide (MSTFA) with a fatty acid methyl esters (FAMEs) retention time ladder with 1% 3,4,5-trimethoxycinnamic acid (TMCA) at 37 °C and incubated for 0.5 h at thermo shaker at 800 rpm. After derivatization, samples were submitted for GC-TOF MS analysis.

#### Data processing

ChromaTOF instrument peak finding and mass spectra deconvolution software version 4.0 was used for peak deconvolution and alignment. BinBase database software (open source; https://code.google.com/p/binbase/) was used for database searching^29^. The features were searched against MassBank of North America (MoNA)^30^ and Lab A’s in-house database.

### Lab B

#### Instrumentation setup

Lab B used an Agilent 7890A/5975 MSD system with the Agilent ZORBAX DB5-MS + 10 m Daugaard Capillary Column (Part number: 122-5532G; Santa Clara, CA) 30 m × 250 µm × 0.25 µm; max temperature: 325 °C; conditioned before use following the manufacturer’s guidelines. The carrier gas was helium (5.0 grade), with a splitless ultra insert and a dimpled 4 mm ID. Samples are injected at 1 µl with using a paused split/splitless injecting mode. The initial temperature was 250 °C, and a 9.02 psi (ON) not fixed setting. The gas saver was open at 3 min. The initial oven temperature was 60 °C which was held for 1 min and ramp to 325 °C at 10 °C/min speed with a 10 min final hold time.

The transfer line of the MSD was set to 290 °C. The MSD was operated at a scan range of m/z 80–600 at 70 eV electron ionization energy. The threshold used was 150. The spectra intensity above 150 was recorded. The MS quad temperature was 150 °C and the MS source temperature was 250 °C. The solvent delay was set at 5.96 min.

#### Samples

The experimental protocols conducted by Lab B were approved by Janssen Research and Development LLC (Spring House, PA, USA) and carried out in accordance with relevant guidelines and regulations. Two batches of NIST (Batch I, n = 6; Batch II n = 6) and pooled human plasma samples (Batch I, n = 6; Batch II, n = 6), each 30 µl, were kept in − 80 °C freezer until analyzed by GC–MS in February, and March of 2018. Post extraction and derivatization, samples were analyzed freshly by GC–MS. The sample preparation protocol followed the same protocol as Lab A samples.

#### Data processing

Lab B processed instrumental raw file (.D) using AMDIS (Agilent, USA) for deconvolution and primary database searching against the Agilent G1676AA Fiehn GC/MS Metabolomics RTL Library (V. 2013, Agilent, USA) and the NIST 14 Mass Spectral Library. The GC/MS features cross all samples were aligned and exported as a text file (.CSV) by Mass Professional Profile 15.0 (Agilent, USA).

### Evaluation of interlaboratory repeatability

The interlaboratory repeatability was evaluated from two aspects: the annotation repeatability and ion intensity. Based on the annotations in each batch of data, the metabolic pathway enrichment analysis and power analysis were performed using MetaboAnalyst 4.0^31^. The statistical protocol for comparison purposes was conducted using Prism 7.0 (GraphPad, San Diego, CA) along with figure generation.
